# Serological Survey of SARS-CoV-2 in Wild Canids in Serbia: First Report in Red Foxes and Golden Jackals

**DOI:** 10.3390/vetsci13040346

**Published:** 2026-04-02

**Authors:** Diana Lupulović, Jelena Maksimović Zorić, Branislav Kureljušić, Nemanja Krstić, Nemanja Jezdimirović, Amer Alić, Bojan Milovanović, Marija Gnjatović

**Affiliations:** 1 Institute for the Application of Nuclear Energy INEP, Banatska 31b, 11080 Zemun, Serbia; nemanja.krstic@inep.co.rs (N.K.); marija.gnjatovic@nitra.gov.rs (M.G.); 2 Institute of Veterinary Medicine of Serbia, Janisa Janulisa 14, 11000 Belgrade, Serbia; jelena.maksimovic@nivs.rs (J.M.Z.); branislav.kureljusic@nivs.rs (B.K.); nemanja.jezdimirovic@nivs.rs (N.J.); bojan.milovanovic@nivs.rs (B.M.); 3 Department of Pathology, Faculty of Veterinary Medicine, University of Sarajevo, Zmaja od Bosne 90, 71000 Sarajevo, Bosnia and Herzegovina; amer.alic@vfs.unsa.ba

**Keywords:** SARS-CoV-2, wildlife, serology, red foxes, golden jackals, One Health, Serbia

## Abstract

Following the COVID-19 pandemic in 2020, there has been growing interest in investigating the transmission routes of SARS-CoV-2 and identifying the potential reservoirs in wildlife. To date, the virus has been detected in a wide range of wildlife animals. SARS-CoV-2 has also been demonstrated to have the ability to infect dogs, raising concerns that other carnivores, such as free-living canids, might serve as a potential source of infection. The aim of this study was to investigate the presence of specific IgG antibodies against SARS-CoV-2 in golden jackals (*Canis aureus*) and red foxes (*Vulpes vulpes*) in Serbia. A total of 165 individuals from ten districts, including the City of Belgrade, were collected and examined from the beginning of August 2024 to the end of March 2025. Of this number, 17 samples were positive (17/165), which represents 10.3% of all the specimens tested. Among 83 red foxes, antibodies against SARS-CoV-2 were detected in 11 animals (13.3%), whereas reactive sera were identified in 6 of 82 jackals (7.3%). The analyses were performed using a commercial multi-species ELISA (IDVet, France), with the results confirmed by an in-house ELISA, previously developed for the detection of COVID-19 in humans and adapted for canids. Our findings confirm that wild animals are indeed exposed to SARS-CoV-2 infection, and that golden jackals and red foxes may represent a potential threat to human health. These results represent the first detection of SARS-CoV-2 seroconversion in wild carnivores in Serbia, highlighting the need for further surveillance and investigation.

## Simple Summary

The virus responsible for COVID-19 can affect multiple animal species, including wildlife. In this study, we tested, by commercial ELISA, blood samples from red foxes and golden jackals in Serbia for IgG anti-SARS-CoV-2 antibodies. Antibodies were detected in both species, with a seroprevalence of 13.3% in red foxes and 7.3% in golden jackals (overall seroprevalence: 10.3%). All the samples were tested in parallel by an in-house adapted ELISA. These results provide the first evidence of SARS-CoV-2 exposure in wild canids in Serbia. Monitoring pathogens in wildlife improves our understanding of disease ecology and supports early recognition of potential zoonotic risks.

## 1. Introduction

Members of the family *Coronaviridae* are ubiquitous worldwide and comprise a group of enveloped, positive-sense single-stranded RNA viruses. Coronaviruses (CoVs) are classified into four genera: *Alphacoronavirus*, *Betacoronavirus*, *Gammacoronavirus*, and *Deltacoronavirus*. Alphacoronaviruses and betacoronaviruses have been confirmed primarily in bats, whereas birds are recognized as the main reservoirs of gamma- and deltacoronaviruses [1]. Coronaviruses are also characterized by a high mutation rate, which contributes to their genetic diversity and adaptive potential. It has long been recognized that coronaviruses can cause respiratory and gastrointestinal diseases in domestic and wild animals. Well-known representatives include canine coronavirus (CCoV), which causes enteric disease in dogs and related species, and canine respiratory coronavirus (CRCoV), which is associated with mild to severe respiratory disorders in dogs [2,3].

In December 2019, a novel coronavirus, severe acute respiratory syndrome coronavirus 2 (SARS-CoV-2), emerged in Wuhan, China, causing coronavirus disease 2019 (COVID-19) [4,5]. The clinical presentation of COVID-19 ranges from mild symptoms such as fever, dry cough, fatigue, and loss of taste or smell to severe disease and fatal outcomes [6]. The rapid spread of the disease overwhelmed healthcare systems, caused substantial economic losses, and led to the implementation of unprecedented preventive measures worldwide, including travel and trade restrictions. Globally, COVID-19 spread at an exceptional pace and resulted in approximately 676 million confirmed human infections and about 6.9 million deaths [7].

Genetically, SARS-CoV-2 is closely related to earlier zoonotic coronaviruses responsible for the severe acute respiratory syndrome (SARS) outbreak in 2002–2003 and the Middle East respiratory syndrome (MERS) outbreak in 2012 [8,9]. However, the epidemiological characteristics of COVID-19 differ considerably from those of previous outbreaks, which were geographically limited. In contrast, COVID-19 exhibited a rapid global spread, reaching pandemic proportions with high morbidity and mortality. It is widely assumed that SARS-CoV-2 originated from wildlife and spilled over to humans at a live animal market in the Chinese province of Hubei. Bats are considered the natural reservoir of the virus, while the intermediate host has not been definitively identified, although the Asian palm civet (*Paradoxurus hermaphrodites*) has been suggested as a potential candidate [10].

SARS-CoV-2 is a single-stranded RNA virus belonging to the genus *Betacoronavirus*. The viral genome encodes four main structural proteins: membrane (M), envelope (E), spike (S), and nucleocapsid (N). The N and S proteins play a key role in inducing the humoral immune response and antibody production [6]. Betacoronaviruses, including SARS-CoV-2, utilize the angiotensin-converting enzyme 2 (ACE2) receptor for host cell entry, a receptor that is present in many animal species [11]. Nevertheless, susceptibility varies among species; for example, experimentally challenged coyotes did not become infected, suggesting that they are not competent hosts for SARS-CoV-2 [12]. Since the beginning of the pandemic, several variants of concern have emerged, including Alpha, Beta, Delta, and Omicron. These variants arise from viral mutations, particularly in the spike protein, and differ in transmissibility, pathogenicity, and their impact on diagnostics and vaccine performance [13].

SARS-CoV-2 is a novel zoonotic virus with significant zoonotic and reverse zoonotic potential. It is assumed that the virus initially spilled over from wildlife to humans and subsequently spread predominantly through human-to-human transmission via close contact and respiratory droplets. However, the virus has also been confirmed in companion animals (dogs and cats), ferrets, and deer, indicating its ability to cross species barriers and cause reverse zoonotic transmission [14,15]. Situations involving close contact between humans and animals, such as pet ownership, hunting activities, and farming, are of particular concern. Notably, SARS-CoV-2 has been detected in white-tailed deer living in proximity to urban areas, suggesting human-to-deer spillover followed by deer-to-deer transmission [16]. Of special interest are minks that are highly susceptible to COVID-19 and develop severe respiratory illness with 35–55% of mortality. Outbreaks on mink farms in Denmark further demonstrated efficient human-to-animal transmission, and viral mutations observed in mink were subsequently associated with spillback and transmission of infection back to humans, which resulted in culling of minks and closing of mink farms [17].

Numerous studies have investigated the presence of SARS-CoV-2 in domestic, wild, and zoo animals to better understand viral origin and identify potential animal reservoirs. According to reports from the World Organization for Animal Health (WOAH), since March 2020, the virus has spread to 36 countries and has been detected in at least 29 animal species across 775 reported events [18]. Carnivores are considered to be at a higher risk of acquiring SARS-CoV-2 infection. Studies in domestic and wild carnivores indicate that infection is often asymptomatic or associated with mild and transient viremia, although mild gastrointestinal and respiratory signs have been reported, particularly in felids. SARS-CoV-2 has been confirmed in wild felids such as lions, tigers, and pumas, as well as in wild canids, including foxes and jackals. In contrast, pigs, cattle, and sheep appear to be resistant to SARS-CoV-2 infection [19,20].

Serological investigation plays a crucial role in detecting past exposure to SARS-CoV-2 in animal populations, particularly because active infection is transient and the virus is difficult to detect by molecular methods after a week, whereas antibodies persist for longer periods. IgM antibodies are detectable for a limited time, followed by the development of IgG antibodies, which may be detected for up to one year, although titers decline significantly after approximately six months [21,22].

In Serbia, the first COVID-19 case was registered on 6 March 2020. According to official data from the Institute of Public Health of the Republic of Serbia, “Dr Milan Jovanović Batut” and the Ministry of Health, a total of 2,459,432 confirmed COVID-19 cases and 17,606 deaths were recorded by January 2023 [23]. Data on SARS-CoV-2 infection in animals in Serbia remain scarce. Nevertheless, several studies have confirmed the presence of SARS-CoV-2 RNA and antibodies in owned dogs and cats [24,25]. Detection of SARS-CoV-2 in domestic carnivores suggests that wild carnivores may also be susceptible to infection [12].

Wild carnivores in Serbia are of particular interest as potential viral sentinels due to increasing population sizes, mostly resulting from the absence of natural predators and the implementation of oral rabies vaccination programs since 2010. According to the Statistical Yearbook of the Republic of Serbia 2023 [26], the estimated population size of red foxes is 53,999 individuals, while the golden jackal population numbers approximately 22,596. Red foxes are widely distributed throughout Serbia, whereas golden jackals are more densely populated in lowland areas, particularly in northern and central regions of the country.

The aim of this study was to assess the exposure of wild canids, specifically red foxes (*Vulpes vulpes*) and golden jackals (*Canis aureus*), to SARS-CoV-2 in Serbia. Blood samples from 165 free-living foxes and jackals were tested for the presence of anti-SARS-CoV-2 antibodies using a commercial ELISA and a parallel in-house ELISA.

## 2. Materials and Methods

### 2.1. Sample Collection and Geographic Distribution

A total of 165 blood samples from red foxes (*Vulpes vulpes*) and golden jackals (*Canis aureus*) were collected for serological testing. Of these, 83 samples originated from foxes and 82 from jackals. The samples were randomly selected based on the available sample volume and hemolysis, ensuring balanced geographical representation across all the study areas. The samples from red foxes and golden jackals were collected from the beginning of August 2024 to the end of March 2025 within the framework of the annual national program for monitoring the effectiveness of oral rabies vaccination in foxes and other wild carnivores, which has been implemented in Serbia since 2011. The program is established by the Veterinary Directorate of the Ministry of Agriculture, Forestry and Water Management of the Republic of Serbia and is conducted in accordance with the Rulebook on the Program of Animal Health Protection Measures for 2024 [27]. All the sampled animals were older than one year, while data on animal sex were not available. The samples were collected from ten administrative districts, including the City of Belgrade, covering a total of 26 municipalities (Table 1).

The blood specimens from red foxes and golden jackals were collected directly from the heart of carcasses by trained hunters within 1–3 days post-mortem. The blood was collected into sterile vacuum blood collection tubes without an anticoagulant. The samples were transported to the Virology Department of the Institute of Veterinary Medicine of Serbia (IVMS) in portable fridges with ice packs to maintain the cold chain during transport. In the laboratory, the blood samples were kept at room temperature to allow spontaneous coagulation and clot retraction. After serum separation, the samples were centrifuged for 10 min at 2500 rpm. The sera were subsequently heat-inactivated at 56 °C for 30 min. Due to variation in post-mortem time, a certain degree of hemolysis was observed in some samples. Therefore, prior to testing, the samples were assessed based on volume and degree of hemolysis. Aliquots of the processed sera were transferred into sterile microtubes and stored at −20 °C until testing.

### 2.2. Serological Screening by Enzyme-Linked Immunosorbent Assay (ELISA)

Serological screening for specific anti-SARS-CoV-2 IgG antibodies was performed using a commercially available and validated ELISA kit, while an in-house ELISA developed at the Institute for the Application of Nuclear Energy (INEP) was applied to selected samples as the comparative method. For clarity, the commercial ELISA (ID Screen^®^ SARS-CoV-2 Double Antigen Multi-Species, IDVet, Innovative Diagnostics, Grabels, France) is hereafter referred to as “IDVet ELISA”, while the adapted assay developed in this study is referred to as “in-house ELISA”.

#### 2.2.1. Commercial ELISA

Laboratory analyses were conducted with a commercial ELISA kit “ID Screen^®^ SARS-CoV-2 Double Antigen Multi-Species” (IDVet, Grabels, France), designed for the detection of antibodies against the nucleocapsid (N) protein of SARS-CoV-2. The assay is based on an indirect ELISA format and is suitable for use in multiple animal species. All the procedures were carried out in accordance with the manufacturer’s instructions.

Briefly, the serum samples were diluted 1:2 in the provided dilution buffer, and 100 µL of the diluted samples, positive control, and negative control were added to microplate wells pre-coated with recombinant SARS-CoV-2 N antigen. The plates were incubated for 45 min at 37 °C. After incubation, the wells were washed five times with 300 µL of wash buffer to remove unbound material. Subsequently, 100 µL of horseradish peroxidase (HRP)-conjugated multi-species secondary antibody was added to each well, followed by incubation for 30 min at room temperature. The plates were then washed five times as described above. Next, 100 µL of tetramethylbenzidine (TMB) substrate solution was added to each well and incubated for 20 min at room temperature in the dark. The enzymatic reaction was stopped by adding 100 µL of stop solution, and the optical density (OD) of each sample was measured at 450 nm using a spectrophotometer Victor 3V PerkinElmer 1420 multilabel counter (Cambridge Scientific, Watertown, MA, USA).

The results were expressed as sample/positive ratio (S/P, %) and interpreted according to the manufacturer’s criteria. The serum samples with S/P values below 50% were considered negative, samples with S/P values between 50% and 60% were classified as doubtful, and samples with S/P values equal to or above 60% were considered positive.

#### 2.2.2. In-House ELISA

An indirect ELISA (iELISA), originally developed for the detection of anti-SARS-CoV-2 IgG antibodies in human COVID-19 patients [28,29] and registered by the Medicines and Medical Devices Agency of Serbia (ELISA SARS-CoV-2 IgG; Certificate of registration No. 515-02-02373-20-003, dated 14 July 2020), was subsequently modified for serological testing of wild canid blood samples. The assay was adapted by replacing the original anti-human IgG secondary antibodies with anti-dog IgG, allowing the quantification of the immune response in wild canids. To confirm the applicability of the secondary anti-dog IgG antibody for fox and jackal sera, a subset of samples previously tested using the commercially validated IDVet ELISA kit was used for comparison.

The in-house ELISA procedure applied in this study is described below:

High-binding 96-well polystyrene microplates were coated overnight at 4 °C with 100 µL per well of recombinant SARS-CoV-2 nucleocapsid (N) protein (ACRO Biosystems, Newark, DE, USA; CAT# NUN-C5227) at a final concentration of 1 µg per well in carbonate–bicarbonate coating buffer (pH 9.0). The plates were washed twice with phosphate-buffered saline (PBS, pH 7.5), stabilized with 200 µL of 1% sucrose per well for 15 min at room temperature, air-dried overnight, and stored at 4 °C until use.

The serum samples were diluted 1:100 in phosphate-buffered saline with Tween-20 (PBST) supplemented with 1% bovine serum albumin (BSA), and 100 µL of each diluted sample was added per well. The plates were incubated for 1 h at 37 °C and washed five times with PBST. Horseradish peroxidase-conjugated rabbit anti-dye IgG secondary antibody (CAT No. ab112828, Abcam, Cambridge, UK), diluted 1:1000 in PBST with 1% BSA, was added at 100 µL per well and incubated for 1 h at 37 °C, followed by washing.

Color development was achieved by adding 50 µL of TMB substrate solution A, followed by 50 µL of TMB substrate solution B per well, incubating for 20 min at 37 °C, and stopping the reaction with 100 µL of sulfuric acid (H_2_SO_4_). Optical density (OD) was measured at 450 nm using a microplate reader (Victor 3V, PerkinElmer, USA).

Positive and negative control sera from red foxes and golden jackals were selected based on their results obtained with the commercial ELISA kit and pooled accordingly. The optical density (OD) value of the negative pool was 0.247, whereas the OD value of the positive pool was 1.142. Blank wells containing 100 µL PBST with 1% BSA were included. The cut-off value was defined as the mean OD of negative sera plus three standard deviations, resulting in a cut-off value of 0.527. Samples with OD values within ±10% of the cut-off (0.474–0.580) were considered doubtful, whereas values below 0.474 were interpreted as negative and values above 0.580 as positive.

### 2.3. Statistical Analyses

Differences in SARS-CoV-2 seroprevalence among species and regions were analyzed by Pearson’s chi-squared test, with 95% confidence intervals (CIs). Ninety-five percent confidence intervals (95% CIs) were calculated to assess the precision of seroprevalence estimates across species and regions. A *p*-value < 0.05 was considered statistically significant. Statistical analyses were performed using Microsoft Excel 2007 (Microsoft Corp., Redmond, WA, USA) and SPSS Statistics version 19.0 (IBM Corp., Armonk, NY, USA).

The cut-off values for positive, negative, and doubtful OD were calculated using R statistical software (version 4.3.2; R Core Team, Vienna, Austria), available at https://www.r-project.org/ (accessed on 15 January 2026).

Agreement between the commercial ELISA and the adapted assay was evaluated using Cohen’s kappa (κ) coefficient [30]. The strength of agreement was interpreted according to commonly accepted criteria (poor: κ < 0.20; fair: 0.21–0.40; moderate: 0.41–0.60; substantial: 0.61–0.80; almost perfect: >0.80).

A multivariate logistic regression model was used to evaluate the association between SARS-CoV-2 seropositivity and selected spatial variables. The dependent variable was serological status (positive/negative), while explanatory variables included species and the distance between sampling locations and the nearest human settlements. Odds ratios (ORs) with 95% confidence intervals were calculated. Statistical analyses were performed using R statistical software (version 4.3.2; R Core Team, Vienna, Austria).

For data visualization, the QGIS 4.0 Development Team (2026) was used (QGIS Geographic Information System, Open Source Geospatial Foundation Project, available at https://qgis.org).

## 3. Results

Serological screening conducted by the commercial ELISA revealed that 17 (10.3%, 95% CI: 5.6–14.9) of the 165 tested red foxes and golden jackals were seropositive for SARS-CoV-2. Among the 83 red foxes, antibodies against SARS-CoV-2 were detected in 11 animals (13.3%, 95% CI: 6.8–22.5), whereas reactive sera were identified in six of 82 jackals (7.3%, 95% CI: 2.7–15). Despite the higher antibody detection rate in red foxes, no statistically significant difference between species was observed (*p* = 0.21).

**Figure 1:**
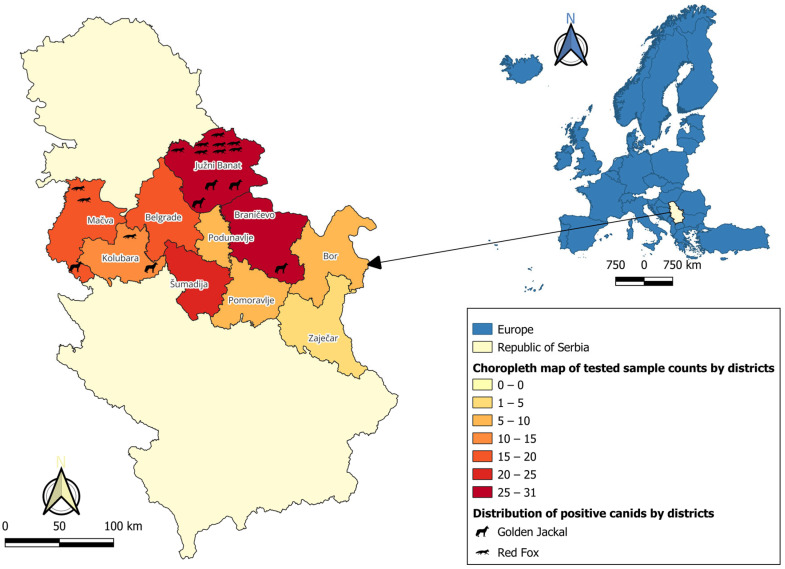
Spatial distribution of tested samples by IDVet ELISA and seropositive wild canids in Serbian districts. Inset: geographic position of Serbia within Europe.

The samples were collected across 10 regions, including the city of Belgrade, but seropositive animals were found in only four districts. Among the seropositive animals, the majority originated from the Južni Banat District, accounting for 64.7% (11/17; 95% CI: 38.3–85.8) of all the positive samples. In the Mačva District, 17.6% (3/17; 95% CI: 3.8–43.4) of positive animals were detected, followed by the Kolubara District with 11.8% (2/17; 95% CI: 1.5–36.4)**.** The Braničevo District contributed 5.9% (1/17; 95% CI: 0.1–28.7) of all the positive samples (Figure 1). Differences in seroprevalence among regions were statistically significant (*p* < 0.001). The distribution of SARS-CoV-2 seropositive wild canids according to species and district tested by commercial ELISA is summarized in the contingency table presented in Table 2.

Notably, the Južni Banat District accounted for 72.7% (8/11) of all the seropositive red foxes and 50.0% (3/6) of all the seropositive jackals, highlighting this district as the main hotspot of SARS-CoV-2 exposure. Overall, the highest seroprevalence was observed in the northern part of Serbia, in the Vojvodina region, indicating both species-specific and regional patterns of exposure among wild canids. The multivariate logistic regression analysis did not reveal a statistically significant association between SARS-CoV-2 seropositivity and distance to the nearest settlement or species (*p* > 0.05).

All the samples were additionally tested with the adapted ELISA. Antibodies against SARS-CoV-2 were detected in 12 of 165 animals (7.3%; 95% CI: 3.8–12.4%), including eight of 83 red foxes (9.6%; 95% CI: 4.3–18.1%) and four of 82 golden jackals (4.9%; 95% CI: 1.3–12.0%).

Using the in-house ELISA, a total of 12 seropositive animals were detected. The majority of the seropositive animals originated from the Južni Banat District, accounting for 50.0% (6/12; 95% CI: 21.1–78.9) of all the positive samples. In the Mačva District, 25.0% (3/12; 95% CI: 5.5–57.2) of the positive animals were identified, followed by the Kolubara District with 16.7% (2/12; 95% CI: 2.1–48.4). The Braničevo District contributed one seropositive animal (8.3%; 1/12; 95% CI: 0.2–38.5). The distribution of SARS-CoV-2 seropositive wild canids according to species and district detected by the in-house ELISA is presented in Table 3.

The comparison between the commercial IDVet ELISA and the in-house ELISA revealed a small number of discordant results, including false positive and false negative samples (Supplementary Table S1). The comparison between the commercial IDVet ELISA and the in-house ELISA revealed a small number of discordant results, including false positive and false negative samples (Supplementary Table S1). Out of the 17 samples identified as positive by the commercial ELISA, 10 were also positive by the in-house ELISA, while seven were not detected by the adapted assay. In addition, two samples were positive only by the in-house ELISA. Scatter plots showing the distribution of ELISA readout values for both assays are presented in Supplementary Figure S1.

The adapted ELISA showed substantial agreement with the commercial assay (Cohen’s κ = 0.66), suggesting comparable performance between the two methods under the conditions of this study.

## 4. Discussion

Surveys describing cases of SARS-CoV-2 infection in animals have so far been widely reported in pets, particularly domestic cats and dogs, across North America, Europe, Asia, and South America [31,32]. However, data on SARS-CoV-2 exposure in wild canids remain scarce, despite the fact that the wildlife species are well-known reservoirs of viral pathogens.

In the present study, we assessed SARS-CoV-2 exposure in free-ranging wild canids, specifically red foxes (*Vulpes vulpes*) and golden jackals (*Canis aureus*) in Serbia. Our results revealed that 17 out of the 165 tested animals were seropositive, corresponding to an overall seroprevalence of 10.3%. Comparable studies conducted in other European countries have demonstrated heterogeneous results. In Croatia, ELISA reactivity was detected in 2.9% of red foxes and 4.6% of the tested golden jackals [20]. In Switzerland, serological exposure to SARS-CoV-2 was confirmed in free-ranging red foxes, with an estimated seroprevalence of 3.1% [33]. In another Swiss study, only one red fox was found to be SARS-CoV-2 positive by qRT-PCR among 246 samples collected from 153 animals housed in zoos [34]. Conversely, a study from Poland did not identify any positive red foxes in either molecular or serological investigations involving 292 hunted animals [35]. We assume that published findings vary significantly depending on the species studied, methodological approaches, sample sizes, and SARS-CoV-2 variants circulating during the pandemic, which was consistent with previous observations [31,36,37].

Furthermore, data from investigations involving other wild canids are similarly limited. Goldberg et al. (2024) [38], during surveillance of wildlife communities across Virginia and Washington, DC, USA, detected SARS-CoV-2 antibodies in 36.4% of tested raccoons. In contrast, several studies failed to confirm SARS-CoV-2 infection in wolves, either in free-ranging populations or zoo-housed animals [39]. Similarly, no SARS-CoV-2-positive coyotes or eastern wolves were detected by RT-PCR in surveillance studies conducted in Ontario and Québec, Canada [3,40].

The fox and jackal samples included in this study originated from ten districts, including the City of Belgrade, while positive samples were detected in only four districts (Južni Banat District, Mačva District, Kolubara District, and Braničevo District). The observed distribution of seropositive animals reflects the epidemiological situation regarding SARS-CoV-2 in the human population in Serbia. Following several waves of COVID-19 between 6 March 2020 and 2022, a period of reduced infection rates was recorded. However, starting from May 2024, SARS-CoV-2 showed moderate fluctuations throughout the season, with a noticeable maximum (103.1 cases per 100,000 population) at the end of September 2024 [41]. Since the animal samples in our study were collected from summer 2024 to spring 2025, we assumed that there is a positive relationship between the proximity of urban areas and seropositivity in wild canids, and these results coincide with the highest peak in the post-COVID-19 season in 2024 in Serbia. Similar associations between urbanization and seropositivity in wildlife have been reported previously [38].

An exceptionally high incidence rate of SARS-CoV-2 in humans was recorded in the Južni Banat District (2646.55 per 100,000 inhabitants), which is consistent with our findings in wild canids from the same region. Significantly, 72.7% of the seropositive foxes and 50% of the seropositive jackals originated from the same district. Our data are in agreement with earlier studies indicating that animals living in close proximity to humans may face an increased risk of exposure [32]. Jackals and foxes are known to approach human habitations, farms, and zoos and likely mix with domestic animals and humans [35,42]. Foxes and jackals are known to transmit numerous pathogens, many of which have zoonotic potential [43]. Red foxes live and move in small groups, while jackals generally live in pairs and have territorial behavior. Jackals are omnivores and scavengers. Both species feed on rabbits, skunks, rodents, and are in close contact with other potential sources and reservoirs of SARS-CoV-2 [12,20].

In the present study, red foxes showed a higher seroprevalence than golden jackals, with antibodies detected in 11 of 83 foxes (13.3%) and six of 82 jackals (7.3%). This is in line with the findings reported by Porter et al. (2022) [12], who showed that experimentally infected foxes developed mild clinical signs and were capable of viral shedding, whereas infected coyotes remained asymptomatic. Such results highlight the importance of further investigations in red foxes, which may represent a potential wildlife host.

Additional investigations suggest that red foxes and golden jackals may be susceptible to SARS-CoV-2 infection because they possess the ACE2 receptor, which enables viral attachment and entry into host cells. Comparative analyses have shown that ACE2 receptors in carnivores share a high degree of similarity with human ACE2, particularly at key amino acid residues involved in spike protein binding [11,44,45]. This conservation indicates that SARS-CoV-2 can potentially interact with ACE2 receptors in canids, including red foxes and golden jackals.

The widespread distribution of ACE2 among mammals, together with the high prevalence of SARS-CoV-2 in the human population during the pandemic, likely facilitated multiple spillback events from humans to animals [11]. In our study, seropositive animals were detected relatively close to human settlements, which may increase opportunities for indirect interactions between wildlife, domestic animals and humans and potentially contribute to further spillover or spillback transmission events. Although the multivariate logistic regression analysis did not identify statistically significant predictors of seropositivity, the relatively short distance between the seropositive animals and nearby settlements suggests that indirect interactions at the wildlife–human interface may still influence exposure.

The expansion of the golden jackal may influence the spatial distribution of other mesocarnivores. Studies have shown that the red fox often behaves as a subordinate species and modifies its realized niche to reduce the probability of direct encounters with golden jackals, resulting in spatial and habitat segregation between the two species [46]. Experimental studies have also demonstrated that red foxes actively avoid jackals and may even abandon profitable food resources to reduce the risk of aggressive interactions [47]. Such behavioral responses may lead to increased use of anthropogenic and peri-urban habitats by foxes. In addition, limited biosecurity implementation on livestock farms in Serbia may facilitate contacts between wildlife and domestic animals [47,48]. In recent years, particularly during the COVID-19 pandemic, an increase in outdoor recreational activities and visits to natural areas has been observed [49]. Such changes in human mobility and behavior can alter interactions between humans and wildlife [50], potentially increasing the probability of zoonotic spillover events when contact between wildlife, domestic animals and humans intensifies [51].

Serological assays are important diagnostic tools for SARS-CoV-2 surveillance, particularly in wildlife studies. Although RT-PCR remains the reference method for confirming active SARS-CoV-2 infection, its application in wildlife surveillance presents several practical limitations. The persistence of viral RNA is transient, the success of detection is highly dependent on the timing of sampling, and the quality of the sample is often inadequate. For these reasons, serological approaches are often more suitable for population-based surveillance and retrospective assessment of viral circulation [21].

Among serological techniques, ELISAs offer several practical advantages. They are technically less demanding, cost-effective, suitable for high-throughput screening, and do not require biosafety level-3 (BSL-3) facilities, unlike virus neutralization tests, which are considered the gold standard but are time-consuming and require trained personnel [21,52].

Many commercial ELISAs for SARS-CoV-2 are based on the nucleocapsid (N) protein. The N protein is highly immunogenic, abundantly expressed during infection, and elicits a strong antibody response, making it a suitable target for serological detection [53,54]. In the present study, we used a commercial multi-species ELISA from Innovative Diagnostics (IDVet), France, based on the N protein. This assay has been widely applied across a broad range of animal species, including cats, dogs, rabbits, bats, wild boars, ferrets, goats, lions, cattle, and horses [55,56,57,58,59]. According to the manufacturer’s Internal Validation Report, the assay demonstrates high diagnostic performance with a reported sensitivity of 99.1% and specificity of 99.1%**.** The test has also been evaluated for potential cross-reactivity with other animal coronaviruses, including canine coronavirus (CCoV), feline coronavirus (FCoV), and bovine coronavirus (BCoV), with no significant cross-reactions reported. The high diagnostic performance of the test has also been supported by previous evaluations [56,58].

All the samples were additionally tested using an adapted in-house ELISA. The lower number of seropositive results obtained with the adapted assay (12/165; 7.3%) compared to the commercial ELISA (17/165; 10.3%) can be explained by methodological differences between the assays. Variations in antigen presentation, assay design, and cut-off determination can influence the classification of samples, particularly those with low antibody levels [60]. Commercial assays are typically validated using large panels of well-characterized samples. In contrast, in-house assays are often developed on smaller sample sets, which may result in reduced sensitivity. Despite these differences, the substantial agreement observed between the assays (Cohen’s κ = 0.66) indicates that the two approaches were broadly comparable. Similar levels of concordance between the commercial and in-house serological assays have been reported previously [61].

As far as we know, this study provides the first evidence of SARS-CoV-2 exposure in wild canids in Serbia. Certain limitations should also be considered. The geographic distribution of the sampled areas was not fully balanced, and the number of collected samples varied among districts. A more uniform sampling strategy would allow a clearer interpretation of the spatial distribution of SARS-CoV-2 exposure.

Finally, further optimization and validation of serological assays for wildlife samples remain essential. As emphasized by Jemeršić et al. (2021) [20], particular attention should be given to sampling methodology, species tested, and sample quality. Wildlife samples are frequently affected by hemolysis and variable storage conditions, which can affect the accuracy of results.

## 5. Conclusions

In conclusion, this study provides evidence of SARS-CoV-2 exposure in wild canids, red foxes and golden jackals, in Serbia, highlighting that wildlife species living close to human environments may be affected by emerging pathogens. Continued surveillance, including a broader range of species and longitudinal monitoring, is essential. Within a One Health framework, integrated monitoring of pathogen circulation at the human–animal–environment interface remains key for understanding of viral dynamics, potential spillover risks and future zoonotic threats.

## Figures and Tables

**Table 1:** Number of blood samples collected by species and districts in Serbia.

District	Species		Total
	Red Fox	Golden Jackal	
Bor	6	4	10
Braničevo	7	21	28
Južni Banat	16	15	31
Kolubara	13	2	15
Mačva	16	3	19
Podunavlje	0	7	7
Pomoravlje	1	5	6
Šumadija	16	9	25
Zaječar	1	3	4
Belgrade	7	13	20
**Total**	**83**	**82**	**165**

**Table 2:** SARS-CoV-2 seropositive wild canids by species and district (IDVet ELISA).

District	Species		Total (%)
	Red Fox (*n*)	Golden Jackal (*n*)	
Južni Banat	8	3	11 (64.7)
Mačva	2	1	3 (17.6)
Kolubara	1	1	2 (11.8)
Braničevo	0	1	1 (5.9)
**Total**	**11**	**6**	**17 (100)**

**Table 3:** SARS-CoV-2 seropositive wild canids by species and district (in-house ELISA).

District	Species		Total (%)
	Red Fox (*n*)	Golden Jackal (*n*)	
Južni Banat	5	1	6 (50.0)
Mačva	2	1	3 (25.0)
Kolubara	1	1	2 (16.7)
Braničevo	0	1	1 (8.3)
**Total**	**8**	**4**	**12 (100)**

## Data Availability

The original contributions presented in this study are included in the article/Supplementary Material. Further inquiries can be directed to the corresponding author.

## Supplementary Materials

The following supporting information can be downloaded at https://www.mdpi.com/article/10.3390/vetsci13040346/s1, Table S1: Comparison of results obtained by the commercial IDVet ELISA and the in-house ELISA; Figure S1: Scatter plots showing the distribution of ELISA readout values for the commercial IDVet ELISA (A) and the in-house ELISA (B).
