# Supplementary material for: Serological Survey of SARS-CoV-2 in Wild Canids in Serbia: First Report in Red Foxes and Golden Jackals
Source: Vet Sci. 2026 Apr 2;13(4):346. doi: 10.3390/vetsci13040346 (PMC13120060; doi:10.3390/vetsci13040346)
Supplement: Supplementary file 1 [file vetsci-13-00346-s001.zip › Supplementary Figure S1.pdf]

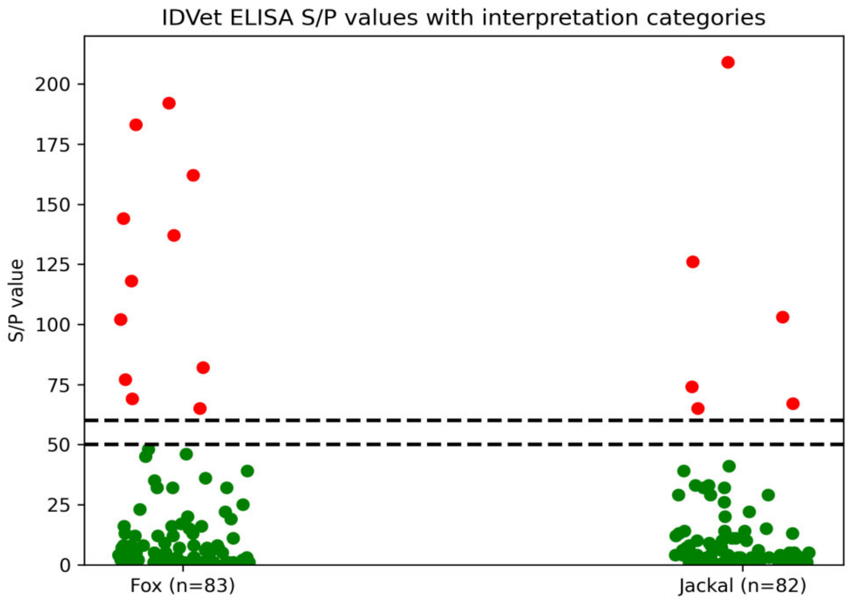

Figure S1 (A): The distribution of ELISA readout values for the commercial IDVet ELISA

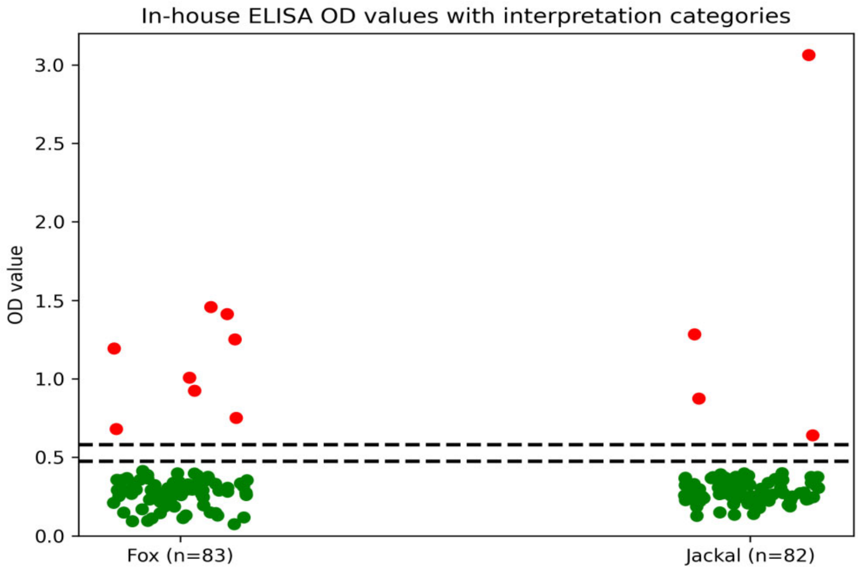

Figure S1 (B): The distribution of ELISA readout values for the in-house ELISA

Figure S1. Scatter plots showing the distribution of ELISA readout values for the commercial IDVet ELISA (A) and the in-house ELISA (B). The horizontal dashed line indicates the cut-off value for seropositivity.
