# Supplementary material for: Serological Survey of SARS-CoV-2 in Wild Canids in Serbia: First Report in Red Foxes and Golden Jackals
Source: Vet Sci. 2026 Apr 2;13(4):346. doi: 10.3390/vetsci13040346 (PMC13120060; doi:10.3390/vetsci13040346)
Supplement: Supplementary file 1 [file vetsci-13-00346-s001.zip › Supplementary Table S1.pdf]

Table S1. Comparison of results obtained by the commercial IDVet ELISA and the in-house ELISA

	IDVet +	IDVet -	∑
In-house +	10	2	12
In-house -	7	146	153
∑	17	148	165
